# Involvement of the retinoic acid signaling pathway in sex differentiation and pubertal development in the European sea bass *Dicentrarchus labrax*

**DOI:** 10.1016/j.heliyon.2019.e01201

**Published:** 2019-02-05

**Authors:** Paula Medina, Ana Gómez, Silvia Zanuy, Mercedes Blázquez

**Affiliations:** a Institut de Ciències del Mar, Consejo Superior de Investigaciones Científicas (CSIC), Barcelona, Spain; b Instituto de Acuicultura de Torre la Sal, Consejo Superior de Investigaciones Científicas (CSIC), Torre la Sal, Castellón, Spain

**Keywords:** Molecular biology, Physiology, Zoology, Developmental biology

## Abstract

Retinoic Acid (RA) is a vitamin A derivative present in many biological processes including embryogenesis, organ development and cell differentiation. The RA signaling pathway is essential for the onset of meiosis in tetrapods, although its role in fish reproduction needs further evidence. This study reports the expression profiles of several genes involved in this pathway during sex differentiation and the first reproductive season in European sea bass (*Dicentrarchus labrax*) gonads. The assessed genes are representative of several steps of the pathway including retinol transport, RA synthesis, nuclear receptors, RA transport and degradation. The study includes a synteny analysis of *stra8*, a tetrapod meiosis gatekeeper, in several taxa. The results show that, these genes were overexpressed during early gonad development and their expression decreased during meiosis progression in males and during vitellogenesis in females. Specifically, a decrease of *cyp26a1*, involved in RA degradation, together with an increase of *aldh1a2* and *aldh1a3*, in charge of RA-synthesis, might ensure the availability of high RA levels at the time of meiosis in males and females. Moreover, the absence of *stra8* in the European sea bass genome, as well as the conserved genomic neighbourhood found in other taxa, suggest a *stra8* independent signaling for RA during meiosis. Taken together, our results might help to better understand the role of RA signaling in teleost gonad development.

## 1 Introduction

Retinoic acid (RA) is the active form of vitamin A, and exerts pleiotropic functions in many biological processes such as differentiation of the nervous system (McCaffery et al., 2003), embryogenesis (Reijntjes et al., 2004), body patterning (Marill et al., 2003), organ and skeletal development (Campo-Paysaa et al., 2008; Spoorendonk et al., 2008) or cell differentiation (Niederreither and Dollé, 2008). More than a century of research has built up a solid framework concluding that the RA signaling pathway is essential for the onset of meiosis in tetrapods (Griswold et al., 2012). Briefly, this pathway involves the initial formation of a complex for retinol (ROL) bloodstream transport, which includes the ROL binding protein-4 (Rbp4). Once in the target tissues, ROL enters the cell by interacting with the trans-membrane receptor Stra6 (stimulated by RA protein 6) (Albalat, 2009; Bowles and Koopman, 2007), expressed in Sertoli cells and other blood organ-barrier tissues (Amengual et al., 2014). Depending on the needs, ROL can either remain bound to Stra6, or be transformed into RA by two tandem oxidations. The first oxidation is mediated by an alcohol dehydrogenase and results in the synthesis of retinal (RAL), whereas the second one is mediated by aldehyde dehydrogenases (Aldhs) (Napoli, 2012). RA is highly unstable and toxic and can either bind to an intracellular protein (Crabp), in order to keep it soluble, or be degraded by Cyp26 enzymes into more polar compounds easier to clear out from the cell (Thatcher and Isoherranen, 2009).

RA signals are transduced by retinoic acid receptors (Rars) and retinoid X receptors (Rxrs), which can hetero- or homodimerize to regulate gene expression. Each receptor consists of three isoforms and their dimerization results in multiple combinations (Chawla et al., 2001). *In vivo*, RA exists mostly as the all-trans isomeric conformation and only trace amounts of the 9-cis isomer can be found. Although both isomers are capable to bind to the ligand binding domain of Rar, Rxr has higher affinity for the 9-cis isomer (Heyman et al., 1992). Moreover, Rxrs are also heterodimeric partners of other nuclear receptors, including thyroid hormone receptor, vitamin D receptor, and peroxisome proliferator-activated receptor (Ppar) (Chawla et al., 2001). The activated ligand/receptor complex binds to RA response elements (RAREs) that regulate the transcription of over 500 genes (Balmer and Blomhoff, 2002). Among them, it is worth mentioning *Stra8* (stimulated by RA gene 8), required for premeiotic DNA replication and the entry in prophase I of germ cells, making it essential for meiosis progression in tetrapods (Griswold et al., 2012).

In mouse, chicken and newt models, the onset of meiosis is induced by the balance between the synthesis and degradation of RA in a species- and sex-specific manner (Bowles et al., 2009; Smith et al., 2008; Wallacides et al., 2009). In mouse, meiosis starts before birth in females and by the time of puberty in males (Koubova et al., 2006), whereas in chicken the involvement of RA in the onset of meiosis has only been proven for females (Smith et al., 2008). In newt, the onset of meiosis in females occurs during larval life while in males it starts during metamorphosis (Wallacides et al., 2009), bringing about changes in the expression of key players of the RA signaling pathway. All these evidences support the role of RA signaling in the timing of the onset of meiosis in tetrapods, however, its role in gonad development and reproduction in fish still needs to be addressed. In this regard, a lack of *stra8* has been reported in several fish species (Pasquier et al., 2016; Rodríguez-Marí et al., 2013). Despite these findings, a *stra8* homolog could be identified in Southern catfish, *Silurus meridionalis* (Dong et al., 2013), and other fish species (Pasquier et al., 2016), suggesting that in fish two different mechanisms, one dependent and the other one independent of the presence of *stra8*, might regulate the RA-mediated entry into meiosis (Feng et al., 2015; Li et al., 2016).

The European sea bass (*Dicentrarchus labrax*) is an excellent model to study the role of the RA signaling pathway in fish reproduction. The gonochoristic status of this marine teleost (Piferrer et al., 2005) allows for the possibility to provide a detailed sex-specific assessment of different components of the pathway during gonad development. The endocrine changes during its reproductive cycle are well known (Carrillo et al., 2015; Mañanós et al., 1997; Prat et al., 1990; Rocha et al., 2009), and different markers of sex differentiation (Blázquez et al., 2008, 2009; Díaz and Piferrer, 2015) and sexual maturation (Blázquez et al., 2017; Crespo et al., 2013) have been identified. This highly valued aquaculture species exhibits a high incidence of precocious puberty, particularly in males, with about 20–30% of them maturing by the end of the first year of life (Begtashi et al., 2004), as opposed to normal maturation that occurs by the end of the second year (Zanuy et al., 2001), influencing negatively growth, animal welfare and final product value (Carrillo et al., 2015; Espigares et al., 2015). The role of RA signaling in European sea bass reproduction is not known, and could represent a source of genetic markers during the attainment of puberty. Recently, a microarray expression profiling study identified 315 genes differentially expressed in testis by the time of meiosis among which, a group of them were involved in the RA signaling pathway (Blázquez et al., 2017). The present study is aimed to describe the expression profiles of several key genes of the RA signaling pathway during sex differentiation and puberty in order to identify possible molecular markers triggering meiosis in the European sea bass.

## 2 Material and methods

### 2.1 Fish and rearing conditions

Freshly fertilized sea bass eggs were obtained from the Institute of Aquaculture Torre la Sal (Castellón, Spain) and immediately transported to our experimental aquaria facilities (ZAE) at the Institute of Marine Sciences in Barcelona (41°23′N; 2°11E). Egg incubation and fish rearing were performed according to standard procedures for sea bass aquaculture (Moretti et al., 1999). Briefly, eggs were placed in 600 L tanks shortly after fertilization (end of March). At four days post hatching (dph), stocking densities were reduced by half and divided into two 600 L tanks in order to create a male-enriched group and a female-enriched group. The male enriched group was obtained following a previously described protocol (Navarro-Martín et al., 2009a), taking advantage of the fact that in this gonochoristic fish species, genotype and temperature contribute to the final sex ratios (Piferrer et al., 2005). Briefly, at 5 dph, the temperature of the rearing water was increased (0.5 ºC/day) up to 20 °C and kept constant until 60 dph when it was brought back to natural conditions for the rest of the experiment. However, it is not possible in this species to obtain a female-enriched population just by thermal manipulations. Therefore, in order to increase the probability to find females in the samplings, fish were fed a commercial pelleted sea bass food containing 10 mg kg^-1^ estradiol (E_2_) using the alcohol evaporation method adapted to this species (Blázquez et al., 1998). This food was administered *ad libitum*, twice a day, for a short 27-day period (between 93 and 120 dph), covering the labile period of sex differentiation (Navarro-Martín et al., 2009b; Piferrer et al., 2005), switching back to untreated food at 120 dph. Since European sea bass does not exhibit any external sexual dimorphism, the generation of male- and female-enriched groups helped us to increase the number of males or females selected at each sampling time, thus reducing the number of animals sacrificed to comply with the 3Rs principle. This treatment was shown to render females with no differences from untreated females in this species (Blázquez et al., 1998; Navarro-Martín et al., 2009a). Moreover, pioneer studies in fish focussed on the clearance kinetics of exogenous sex steroids showed that, regardless of the route of administration, the hormones were eliminated from the body in a matter of days (reviewed in Piferrer, 2001). Fish were reared and sacrificed according with the Spanish regulations (Royal Decree Act 53/2013), the European legislation concerning the protection of vertebrates used for experimental and other scientific purposes (2010/63 EU) and in accordance with the Society for Study of Reproduction's specific guidelines. The animal facility is approved for animal experimentation (REGA number ES080190036532) and the experimental procedures were approved by the Spanish National Research Council (CSIC) Ethical Committee within project AGL2011-28890). All the recommendations were followed to reduce animal suffering.

### 2.2 Gonad development and sampling intervals

**Fig. 1:**
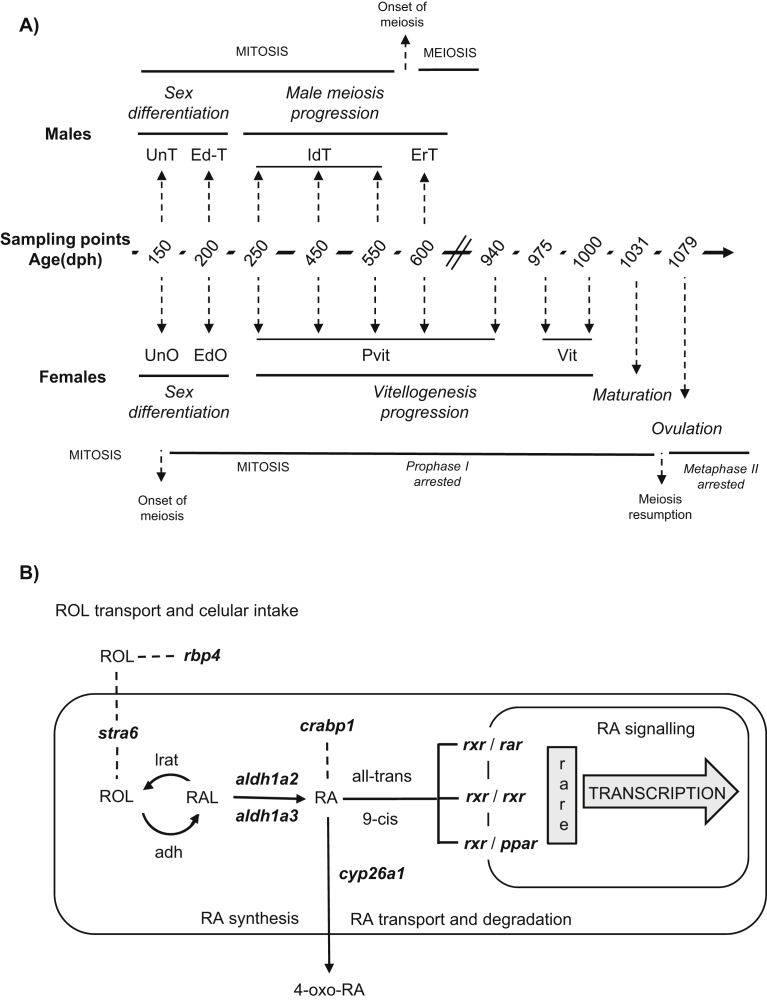
A) Experimental design depicting the sampling time points represented as days post hatching (dph) during the different gonad developmental stages covered by the study. Vertical arrows link the different sampling points with their developmental stage and their corresponding period (in italics) during puberty in European sea bass. Abbreviations: undifferentiated ovary (UnO), undifferentiated testis (UnT), early differentiated testis (EdT), immature differentiated testis (IdT), early recrudescent testis (ErT), previtellogenesis (Pvit), vitellogenesis (Vit), maturation (Mat) and ovulation (Ovu). B) Schematic representation of the retinoic acid signaling pathway. Genes for which their expression levels were assessed in the study are represented in bold. Dotted line stands for binding protein interactions. Abbreviations: retinol (ROL), retinoid binding protein 4 (*rbp4*), stimulated by retinoic acid 6 (*stra6*), retinal (RAL), lecithin retinol acyltransferase (*lrat*), alcohol dehydrogenase (*adh*), aldehyde dehydrogenase 1a2 (*aldh1a2*), aldehyde dehydrogenase 1a3 (*aldh1a3*), retinoic acid (RA), cellular retinoid binding protein 1 (*crabp1*), polar molecules product of RA degradation (4-oxo-RA), retinoid receptor x, isoform a (*rxra*), retinoic acid receptor, isoform a (*rara*), peroxisome proliferator-activated receptor, isoform g (*pparg*), retinoic acid response element (rare).

Males from the male-enriched group were sampled from August 2012 (150 dph) to November 2013 (600 dph) including different representative stages of sex differentiation and early puberty. Females from the E_2_-treated group were sampled from August 2012 (150 dph) to March 2015 (1079 dph), for a total of 11 sampling points (see Fig. 1A for experimental design). In each sampling, fish were anesthetized in 0.2% phenoxyethanol, assessed for standard length (±0.1 cm) and weight (±0.001g) and sacrificed by severing their spinal cord. Gonads were removed and a fraction of each one was fixed in 4% buffered paraformaldehyde (PAF) for histological assessment of the developmental stage. The rest was snap-frozen in liquid nitrogen and kept at -80 °C for further gene expression analysis.

### 2.3 Histological procedures

Fixed gonads were dehydrated in an increasing ethanol series, embedded in Histoplast (Thermo Scientific), cut at 5 μm, and stained with haematoxylin-eosin. The exact developmental stage was determined by light microscopy following the criteria described in (Begtashi et al., 2004; Espigares et al., 2015) for testes and in (Asturiano et al., 2000; Mayer et al., 1988) for ovaries. Briefly, four developmental periods were defined (Fig. 1A), first sex differentiation which includes the transition from undifferentiated gonads until the histological visualization of a testis (early differentiated testis) or an ovary (early differentiated ovary). Since no histological sex-related features were observed in the undifferentiated gonads, the assignment of sex was based on *cyp19a1a* and *amh* expression levels (Supplemental Fig. S1). *cyp19a1a* has been used as molecular marker for sex assignment in the European sea bass with higher expression in future females than in males (Blázquez et al., 2008, 2009; Díaz and Piferrer, 2015), while in other teleosts the opposite pattern has been shown for *amh* (Fernandino et al., 2008). Second, male meiosis progression which includes the transition from an immature differentiated testis to an early recrudescent testis. Third, vitellogenesis progression which covers the transition from a previtellogenic ovary to the onset of vitellogenesis. Fourth, final ovarian maturation which includes the last physiological transformations in the oocyte prior to spawning and ranges from maturation to ovulation. A brief resumption of meiosis occurs in oocytes by the time of final ovulation and becomes arrested again until fertilization, when meiosis is finally completed with the extrusion of the second polar body (Lubzens et al., 2010; Mayer et al., 1988). Six males and six females in the same stage of gonad development were selected at every sampling point up to 600 dph. From 600 dph onwards, only females (n = 6 at each sampling point) were taken.

### 2.4 RNA extraction and cDNA synthesis

Total RNA was extracted with TRIzol® (Invitrogen™) following the manufacturer's instructions and eluted in nuclease free water. RNA concentration and quality were measured using Nanodrop spectrophotometer (Thermo Scientific, Wilmington, DE) and checked in 1% agarose gel electrophoresis. In order to compare between samples, total RNAs (200 ng) were transcribed into cDNA using Superscript III (Invitrogen) and random hexamers according to the manufacturer's protocol. The resulting cDNAs were used as a template to amplify the genes of interest using real-time quantitative polymerase chain reaction (qPCR).

### 2.5 Primer design and quantitative real-time PCR assays

To ease the understanding of the RA signaling pathway, the studied genes were classified into four different groups, according to their putative role along the pathway (Fig. 1B); a) a ROL transport and cellular intake-related group including the stimulated by retinoic acid gene 6 (*stra6*) and the retinoid binding protein 4 (*rbp4*); b) a group of RA synthesis-related genes including the aldehyde dehydrogenase family1a2 (*aldh1a2*) and the aldehyde dehydrogenase family 1a3 (*aldh1a3*); c) a group of genes involved in RA-signaling that encoded for nuclear receptors, including retinoic acid receptor alpha (*rara*), retinoid receptor alpha (*rxra*), and peroxisome proliferator-activated receptor gamma (*pparg*); d) a group of genes involved in RA transport and degradation that included the retinoic acid binding protein 1 (*crabp1*) and the RA degrading gene cytochrome P450 26a1 (*cyp26a1*). In addition, gonadal aromatase (*cyp19a1a*) and the anti-müllerian hormone (*amh*) were used as markers of sex differentiation at early developmental stages. Gene sequences were obtained from annotated public databases, or predicted with a blast search on the European sea bass genome (Tine et al., 2014). Primers were designed using Primer3 software (http://primer3.ut.ee), all of them featured similar melting temperatures and were located in intron-exon boundaries to check for possible genomic contamination. Amplification efficiencies (*E*) were calculated using a linear regression between a cDNA dilution series of the mean cycle quantification (Cq) plotted against the log amount input cDNA, where *E* = 10^(−1/slope)^. Regression slopes resulted in values around -3.3 and *E* values around 2.0. In all cases, linear correlations between the mean Ct and the cDNA dilution were equal or higher than 0.96 (Supplementary table S1).

Amplifications (qPCR) were performed with a 7300 real-time PCR System (Applied Biosystems) using 2 μl of EvaGreen qPCR Mix Plus (Cultek Molecular Bioline, Madrid) according to the manufacturer's instructions. The reaction included 500 nM of each primer, and 1 μl of a 1:10 dilution of first strand cDNA as template in a 10μl final volume. Samples were run in triplicate in optically clear 96-well plates and for each plate, technical and biological replicates were added in order to compare between runs. Cycling parameters were 50 °C for 2 min, 95 °C for 10 min, 40 cycles at 95 °C for 15 s and 60 °C for 1 min. Finally, a dissociation step at 95 °C for 15 s, 60 °C for 1 min and 95 °C for 15 s was included. Expression values were calculated using the qBase quantification method (Hellemans et al., 2007). Briefly, this method is a modification of the ΔΔCt classic model that takes into account multiple reference genes and gene specific amplification efficiencies, as well as the errors on all measured parameters along the entire calculation. Furthermore, an inter-run calibration algorithm allows for the correction of differences among runs. With this purpose, two reference genes, *18S rRNA* and *ef1a,* previously shown to be the most stable housekeeping genes in this species (Mitter et al., 2009) and routinely used in our group for studies of gonad development (Rocha et al., 2009) were used to normalize inter-run measurements (Further information is available in Supplementary methodology). For the first time in this species, the use of a correction factor calculated from the expression of two reference genes allowed us to compare all the studied genes in two different tissues (testis and ovaries), during different developmental stages that otherwise would not have remained stable along the experiment.

### 2.6 European seabass *stra8* search and sequence analysis

To identify the presence of *stra8* in European sea bass, a protein query was made using *stra8* sequences from different vertebrates (Supplementary table S2). The sequences were used as blast queries against the sea bass genome database to check whether *stra8* was annotated in this species. Synteny analysis of *stra8* in tetrapods and fish was carried out with available information from genome assemblies found in Ensembl (http://www.ensembl.org) and from the European sea bass genome database (Tine et al., 2014). The genomic neighbourhood was established including 10 of the most conserved genes surrounding *stra8* in tetrapods (Supplementary table S3). In order to check for the presence of this well conserved genomic neighbourhood in fish, a search for the genomic location of each gene was conducted in other teleosts.

### 2.7 Statistical analysis of data

Statistical analyses were performed with Statistica (StatSoft, Tulsa, OK, USA) software. All data were previously tested for normality (Shapiro-Wilk test) and log transformed when needed to ensure the homoscedasticity of variances. Variation of relative gene expression within sexes was assessed by a two-way ANOVA and was expressed as normalized relative quantities (Hellemans et al., 2007). Tukey post hoc test (multiple comparisons) was used to check for statistical differences (*P* ≤ 0.05).

## 3 Results

### 3.1 Gonad development

**Fig. 2:**
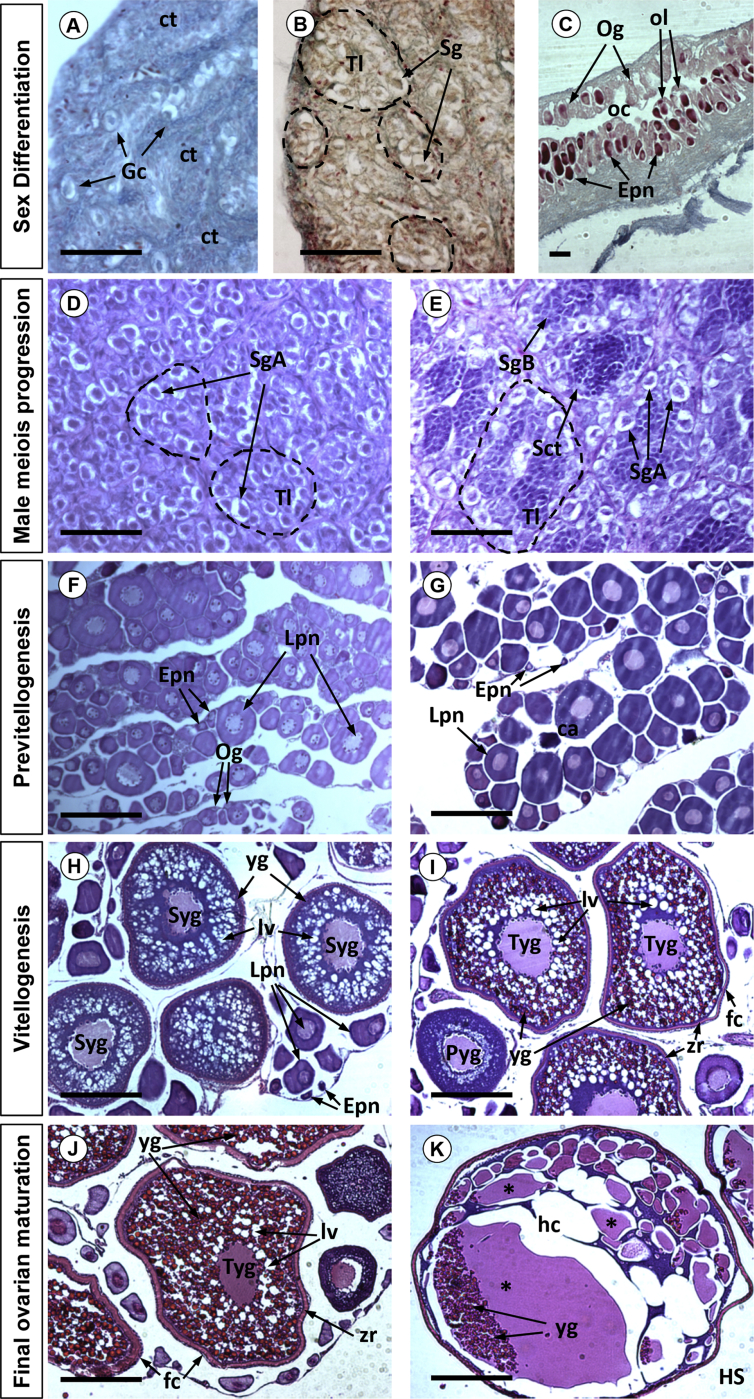
Histological sections of European sea bass gonads at different stages of development. The stages include sexual differentiation, covering from an undifferentiated gonad- 150 dph with the presence of isolated germ cells immersed in connective tissue (A) to an early differentiated testis- 200 dph with the organisation of the gonad in testicular lobules (B) or an early differentiated ovary-200 dph with a central ovarian cavity and the organisation of ovarian lamellae (C). Male meiosis progression, ranging from an immature differentiated testis- 250 dph (D) to a recrudescent testis-600 dph (E). Vitellogenesis progression covering from a previtellogenic ovary 250 dph (F–G) to a full vitellogenic ovary-940 dph. (H–I). Final ovarian maturation including the transition between a mature ovary-1031 dph with the prevalence of oocytes in tertiary yolk granule stage (J) and ovulation-1079 dph with the appearance of oocytes in hyaline stage (K). Dotted lines encircle individual testicular lobules. Abbreviations: germ cell (Gc), connective tissue (ct), spermatogonia (Sg), type A spermatogonia (SgA), type B spermatogonia (SgB), type I spermatocytes (Sc), Tl (testicular lobule), ovarian cavity (oc), ovarian lamellae (ol), oogonia (Og), early perinucleolar oocyte (Epn), late prinucleolar oocyte (Lpn), primary yolk granule stage oocyte (Pyg), secondary yolk granule stage oocyte (Syg), tertiary yolk granule stage oocyte (Tyg), hyaline stage oocyte (HS), yolk granule (yg), lipid vesicle (lv), zona radiata (zr), follicular cells (fc), hydrated cytoplasm (hc). Asterisks represent coalesced yolk granules. In all photographs, the scale bar equals 50μm.

The male-enriched group resulted in a 70% male: 30% female ratio after the temperature treatment whereas an all-female group resulted after E_2_ treatment. At 150 dph, total body length (TL) ranged between 7.3 cm and 9.9 cm. The histological study revealed a common *Undifferentiated stage* in testis and ovaries (Fig. 2A). Sex-related differences appeared at about 200 dph; at a TL 10.1–10.7 cm in males and 10–12.4 cm in females. *Early differentiated testes* (Fig. 2B) showed well-developed testicular lobules with spermatogonia as the predominant cell type and *Early differentiated ovaries* (Fig. 2C) were organised in ovarian lamellae with oogonia (8–16 μm diameter) and perinucleolar oocytes (30 μm diameter). As gonad development progressed, *immature differentiated testes* (250, 450 and 550 dph; TL 11–17 cm) showed spermatogonia A as a predominant cell type (Fig. 2D). Testicular development was assessed until *early recrudescent testes* (600 dph; TL 11.5–19.5 cm) showing cysts of spermatogonia A and B and a small number of type I spermatocytes (Fig. 2E).

In females, the *early differentiated* stage was followed by a long *previtellogenic stage* that lasted up to the third year of life and was assessed at five sampling points (250, 450, 550, 600, 940 dph; TL 10.7–31.5 cm). The *previtellogenic ovary* exhibited isolated oogonia and a few oocytes in the transition from early to late perinucleolar stage (60–120 μm diameter; Fig. 2F–G). The *vitellogenic ovary* (975 and 1000 dph; TL 23–38.5 cm), was characterized by an increase in the number of late perinucleolar oocytes in secondary growth phase (>120 μm diameter) and the presence of oocytes at primary yolk granule stage (260–440 μm diameter; Fig. 2H). In addition, oocytes at secondary yolk granule stage (440–530 μm diameter) and some at tertiary yolk granule stage (530–800 μm diameter) were also present (Fig. 2I). At the *oocyte maturation* stage (1031 dph; TL 23.2–30.5 cm), ovaries showed a prevalence of oocytes at tertiary yolk granule stage (Fig. 2J). *Ovulation* (1079 dph; TL 24.7–34 cm) was marked by the presence of oocytes at tertiary yolk granule stage and hyaline stage (1100–1150 μm diameter; Fig. 2K).

### 3.2 Molecular markers of early sex differentiation (*cyp19a1a* and *amh*)

Gonadal aromatase (*cyp19a1a*) and (*amh*) were used as proxies to discriminate between males and females at 150 dph, before histological sex differentiation was completed. At 150 dph, the low *cyp19a1a* expressors found in the temperature-treated group were unequivocally classified as males and the high expressors as females (Supplementary Fig. S1A). These values were correlated with high *amh* and low *amh* levels, respectively (Supplementary Fig. S1B). It is worth mentioning that only fish with low *cyp19a1a* and high *amh* expression levels were classified as males (undifferentiated males) and used for the analysis. All fish in the E_2_-treated group exhibited high *cyp19a1a* and low *amh* expression and were classified as females. This was confirmed at the end of the experiment since a 100% female population was found in that group (not a single male was found in any of the samplings performed during the three years that lasted the experiment). In agreement with a previous study (Blázquez et al., 2017), we also found a sharp decrease of *amh* during the transition between late differentiated and early recrudescent testis (Supplementary Fig. S2) that was used to confirm meiosis initiation.

### 3.3 Gene expression patterns of RA signaling pathway genes during development

#### 3.3.1 ROL transport and cellular intake-related genes (*rbp4* and *stra6*)

**Fig. 3:**
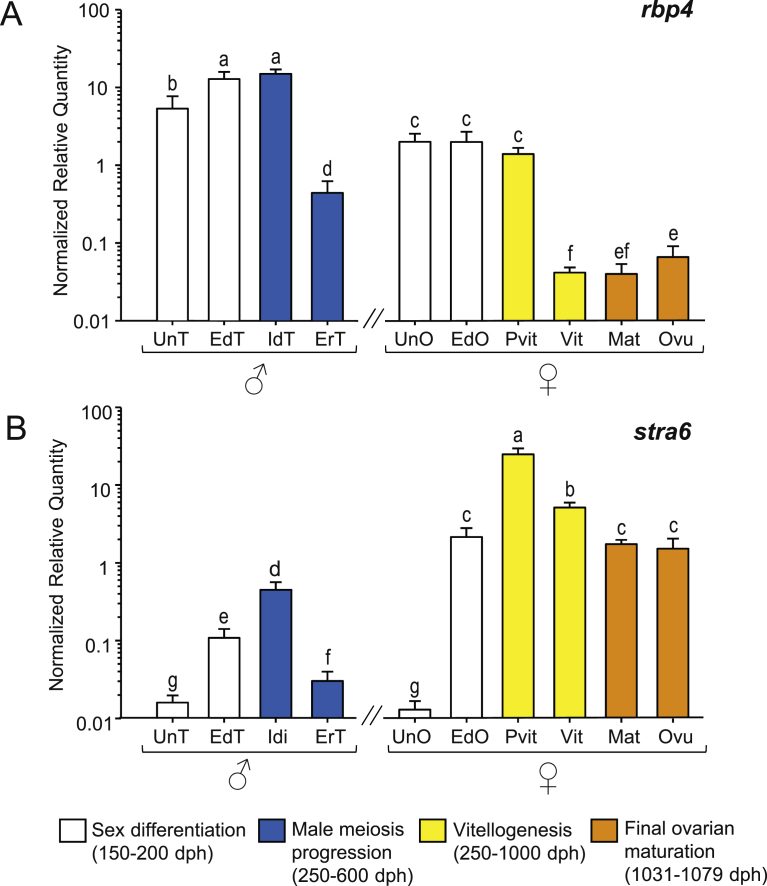
Expression patterns of the RA signaling pathway transport and cellular intake-related genes, (A) *rbp4* and (B) *stra6* in male and female European sea bass gonads. White bars represent gonad differentiation which includes undifferentiated testis (UnT) or undifferentiated ovary (UnO), and early differentiated testis (EdT) and ovary (EdO). Blue bars represent male meiosis progression i.e., the transition from immature differentiated testis (IdT) to early recrudescent testis (ErT). Yellow bars represent the vitellogenic process and include the transition from previtellogenesis (Pvit) to vitellogenesis (Vit). Orange bars represent final maturation and include maturation (Mat) and ovulation (Ovu). Samples were analysed by quantitative real-time fluorescent PCR. Expression data are shown as the normalized relative quantities + SEM of six samples run in triplicate and plotted on a logarithmic scale for easier visualization. Values were normalized to those of the constitutively expressed *18S* rRNA gene corrected with the expression of *ef1a* amplified from the same reverse transcribed template. Different letters denote significant differences after a Tukey test (*p* < 0.05).

Higher *rbp4* levels were generally found in males than in females with the highest values during differentiation and in immature differentiated testis. At the end of this stage, coinciding with early recrudescence, a significant decrease was observed (Fig. 3A). Conversely, no changes during sex differentiation and previtellogenesis were found in females, although a significant decrease occurred at vitellogenesis, remaining low during maturation and slightly increasing by the time of ovulation (Fig. 3A).

Regarding *stra6*, mRNA levels were higher in females than in males, opposite to what has been shown for *rbp4*, except in histologically undifferentiated gonads where the lowest levels were found for both sexes (Fig. 3B). A significant increase in *stra6* was found during gonad differentiation (*P* < 0.001) reaching up to a hundred fold in ovaries. Expression peaked in immature testis and previtellogenic ovaries, followed by a decrease in early recrudescent testis, coinciding with the onset of male meiosis, and throughout vitellogenesis, reaching the lowest values during maturation and ovulation.

#### 3.3.2 RA synthesis-related genes (*aldha1a2*, *aldh1a3*)

**Fig. 4:**
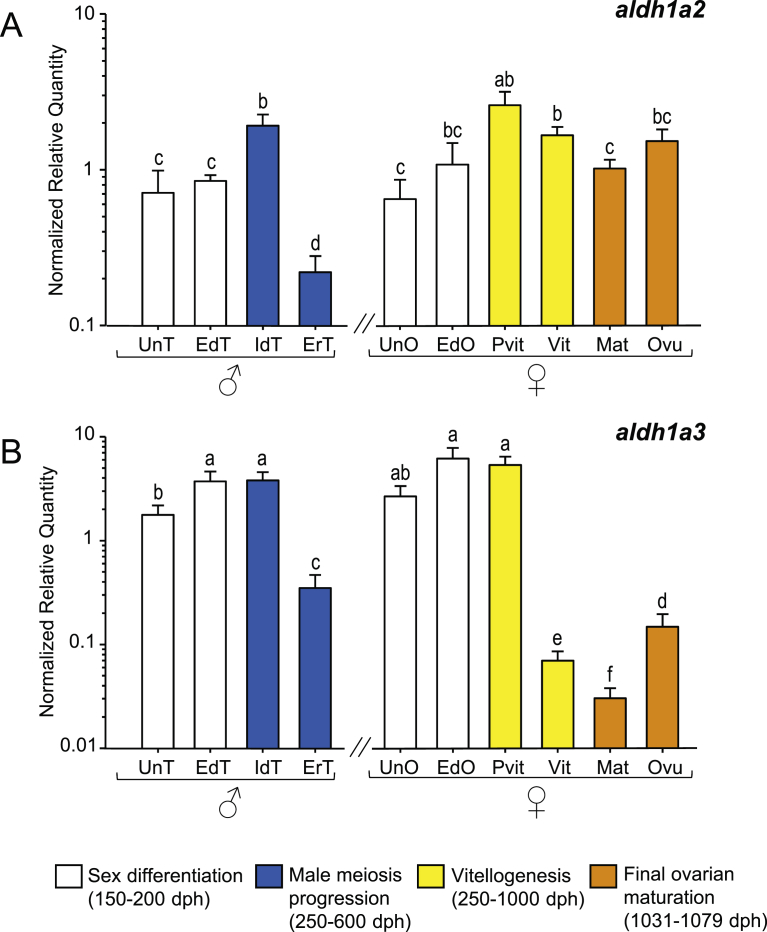
Expression patterns of RA synthesis related genes, (A) *aldh1a2* and (B) *aldh1a3* in male and female European sea bass gonads. Check legend from Fig. 3 for further details.

Similar expression patterns were found in both *aldhs*, progressively increasing from undifferentiated gonads until immature differentiated testis and previtellogenic ovaries (Fig. 4A–B). A significant decrease of the two *aldhs* was found in early recrudescent testis. In females, *aldhs* decreased during vitellogenesis and maturation and increased back again during the transition between maturation and ovulation especially for *aldh1a3*.

#### 3.3.3 RA nuclear receptor genes (*rara*, *rxra*, *pparg*)

**Fig. 5:**
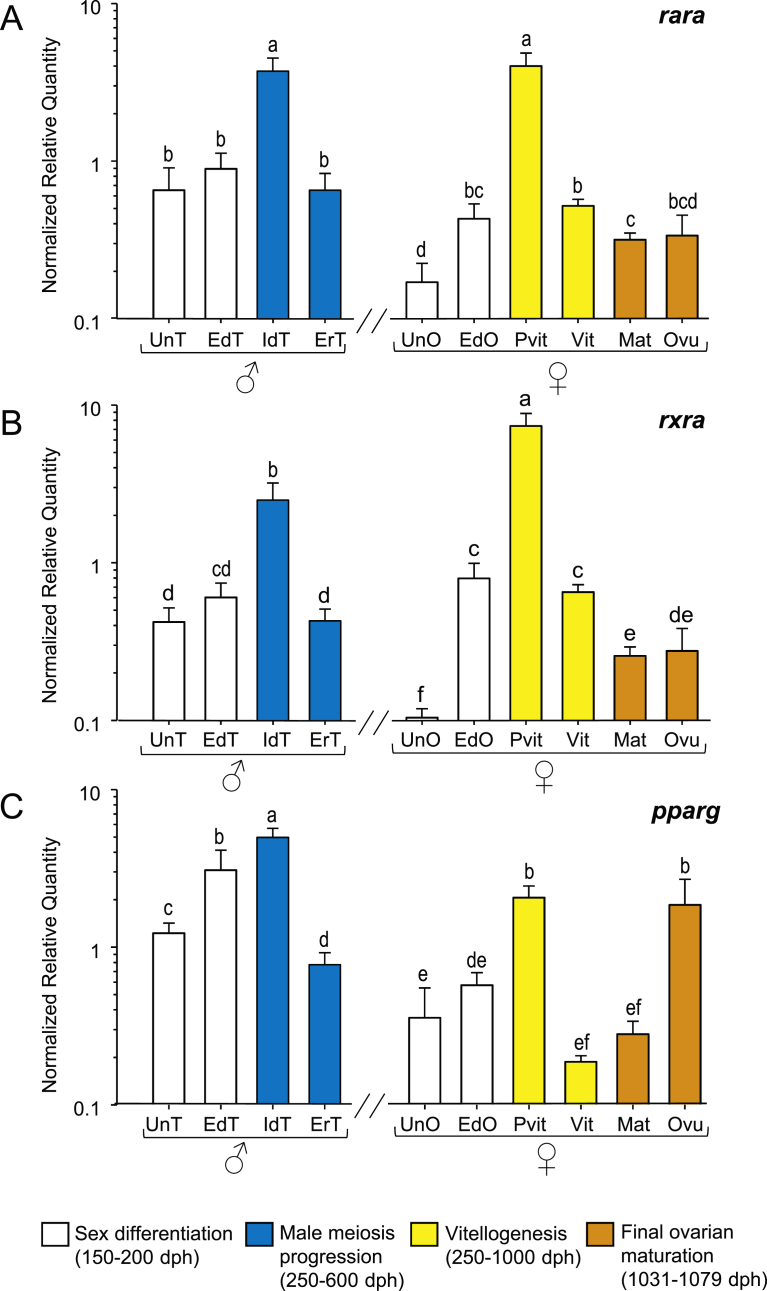
Expression patterns of RA nuclear receptor genes, (A) *rara*, (B) *rxra* and (C) *pparg* in male and female European sea bass gonads. Check legend from Fig. 3 for further details.

Undifferentiated females exhibited lower *rara* and *rxra* expression than males at the same stage while similar levels were found in both sexes by the end of gonad differentiation (Fig. 5A–B). In males, the highest *rara, rxra* and *pparg* levels were found in immature differentiated testis followed by a decrease during early recrudescence, returning to values close to those found during sex differentiation. In females, *rara, rxra* and *pparg* were highest in previtellogenic ovaries with a marked decrease during vitellogenesis and maturation and remaining low during ovulation with the exception of *pparg* that increased again by the time of ovulation to similar levels than those found in previtellogenic ovaries (Fig. 5C).

#### 3.3.4 RA transport- and degradation-related genes (*crabp1*, *cyp26a1*)

**Fig. 6:**
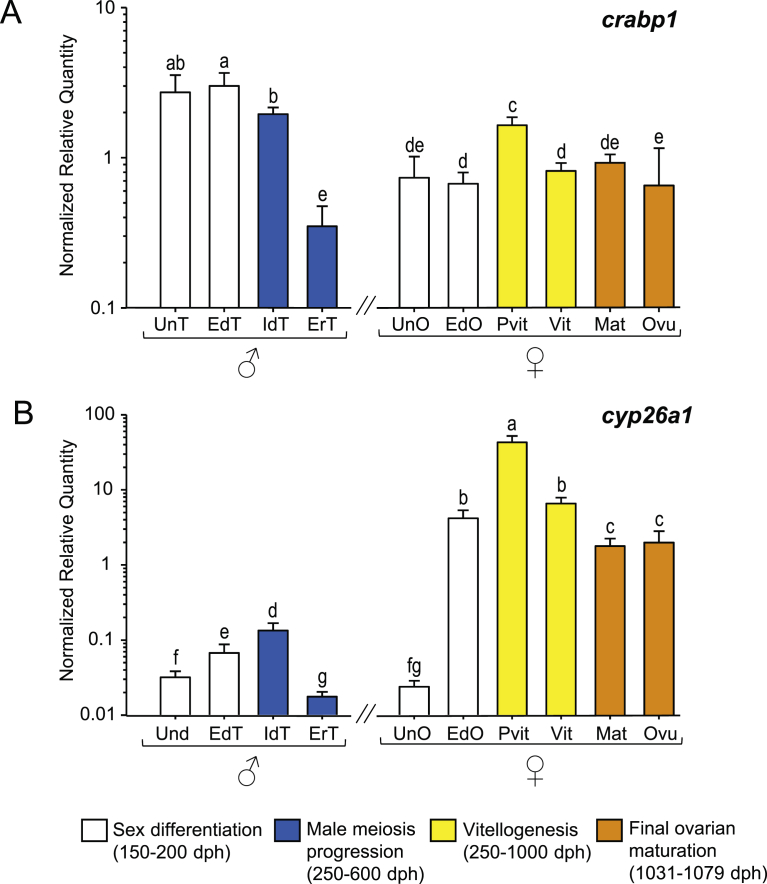
Expression patterns of RA transport and degradation related genes, (A) *crabp1* and (B) *cyp26a1* in male and female European sea bass gonads. Check legend from Fig. 3 for further details.

No changes in *crabp1* expression levels were found during male and female gonad differentiation, although a significant decrease occurred in early recrudescent testis. The highest values of *crabp1* were found in previtellogenic ovaries, that decreased again during vitellogenesis and remained low during maturation and ovulation (Fig. 6A).

The expression of *cyp26a1* was characterized by an increase at the end of gonad differentiation in both sexes (Fig. 6B). Interestingly, in females, this increase reached up to a hundredfold in previtellogenic ovaries whereas in males a marked *cyp26a1* decrease was found in early recrudescent testis. Furthermore, vitellogenesis progression was characterized by a decrease in the expression of *cyp26a1* whereas no significant changes were found during maturation and ovulation.

### 3.4 European sea bass *stra8* sequence search

**Fig. 7:**
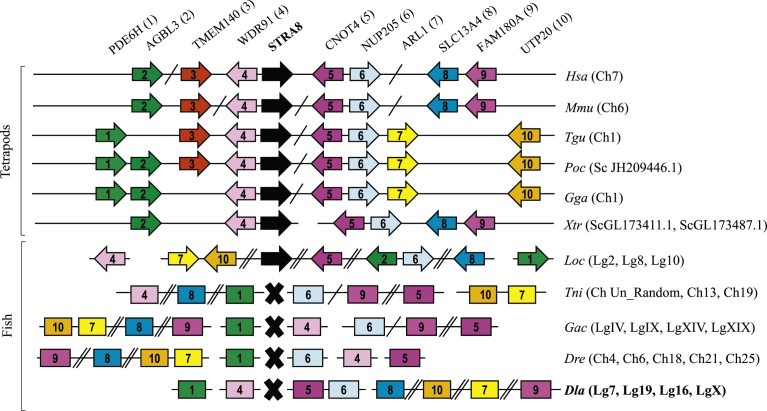
Synteny analysis of *Stra8* gene in tetrapods and fish. Gene name abbreviations are shown on top of the figure and a number between brackets is used to identify each gene. Continuous horizontal lines represent regions of the same chromosome. Simple transverse bars between two genes indicate they are not adjacent. Double transverse bars represent large chromosome regions between two genes. Each gene is represented by a coloured arrow with its identifying number. The direction of the arrow indicates the orientation of the transcription unit with respect to *stra8*. The chromosome (Ch) number, ligation group (Lg) or scaffold (Sc) number in which each gene cluster is located appears indicated between brackets. The black cross represents the absence of the gene. In the particular case of fish where *stra8* has not been found in their genomes, the different neighbouring genes were represented with a box, instead of an arrow, and thus no orientation is depicted in the figure. Abbreviations: *Hsa* (*Homo sapiens*; Human), *Mmu* (*Mus musculus*; Mouse), *Tgu* (*Taenopygia guttata*; Zebra Finch), *Poc* (*Pelodiscus oculatus*; Chinese softshell turtle), *Gga* (*Gallus gallus*; Chicken), *Xtr* (*Xenopus tropicalis*; Frog), *Loc* (*Lepistosteus oculatus*; Spotted gar), *Tni* (*Tetraodon nigroviridis*; Pufferfish), *Gac* (*Gasterosteus aculeatus*; Stickleback), *Dre* (*Danio rerio*; Zebrafish), *Dla* (*Dicentrarchus labrax*; European sea bass). Information about *Stra8* gene accession numbers, and genome assemblies used, is compiled in Supplementary table S2 while information about the genes conforming *Stra8* genomic neighbourhood appear included in Supplementary table S3.

The *stra8* genomic neighbourhood was well conserved among tetrapods (Fig. 7), however, in available fish genomes, *stra8* was only found in the ancient holostei spotted gar (*Lepisosteus oculatus*), showing a non-conserved synteny when compared to tetrapods. It is worth noting that the spotted gar appeared before the emergence of teleosts and therefore, prior to the teleost-specific whole genome duplication. No significant hits for European sea bass *stra8* could be found using several available vertebrate sequences after a blast search against the genome database. Moreover, the conserved genome arrangement found in tetrapods was scattered in different chromosomes or chromosomal regions in fish genomes (Fig. 7). Similarly, no *stra8* hits were found in any of the modern teleosts for which the genome has been sequenced including tetraodon (*Tetraodon nigroviridis*), stickleback (*Gasterosteus aculeatus*), and zebrafish (*Danio rerio*).

## 4 Discussion

The present study provides the first comprehensive analysis of the expression patterns of several key components of the RA signaling pathway during gonad differentiation and the onset of puberty in male and female European sea bass. In the particular case of females, the study covers until their first maturation at the end of the third year of age when meiosis I was completed. In agreement with previous studies, the use of *cyp19a1a* allowed us to discriminate between males and females before histological sex differentiation has occurred (Blázquez et al., 2008, 2009; Díaz and Piferrer, 2015). Regarding *amh*, this is the first time that it is successfully used as molecular marker for testicular differentiation in this species and is confirmed as a marker of the onset of meiosis previously reported in the European sea bass (Blázquez et al., 2017). Altogether, the results can be of particular relevance for other teleosts since the availability of retinoids in the eggs is essential for proper embryonic development in fish (He et al., 2009; Lubzens et al., 2010). Furthermore, our results suggest the involvement of RA in the timing of male and female meiotic initiation, probably using an *stra8* independent signaling pathway (Koubova et al., 2014).

In mammals, retinoids are transferred from the dam to the fetus via the placenta (Quadro et al., 2004), whereas in oviparous vertebrates, they remain stored in the egg during vitellogenesis (Levi et al., 2008). ROL binds to Rbp4 and is transported from the liver to the peripheral tissues where it binds to the membrane receptor Stra6 and gets incorporated inside the target cells (Kawaguchi et al., 2007). In mice, *Stra6* is expressed in Sertoli cells and provides nutrients, including vitamin A, to support spermatogenesis (Bouillet et al., 1997). In fish, in addition to the liver, *rbp4* expression has also been found in gonads, including testicular Sertoli cells (Funkenstein, 2001) and ovary (Levi et al., 2008; Sammar et al., 2001), suggesting that retinoid transport and storage are important for reproduction. Our results show highest *rbp4* and *stra6* expression levels in late differentiated testis, suggesting a need of ROL within the testicular environment in the stages previous to the onset of meiosis, time when the expression of *rbp4* and *stra6* decreased again, probably due to the exhaustion of ROL reserves by their transformation into RAL and RA.

In fish ovaries, ROL can remain bound to Stra6, or become transformed into RAL, the main retinoid in fish eggs (Devlin and Nagahama, 2002; Irie and Seki, 2002; Lubzens et al., 2010), entering the oocyte together with vitellogenin as part of the HDL plasma fraction (Lubzens et al., 2010). In this study, the high *rbp4* and *stra6* levels found in previtellogenic ovaries, just before the start of vitellogenesis, suggest that an increase of intracellular ROL transport and uptake is required to supply the needs of the oocyte during this process. A similar result was found in zebrafish where the expression of *stra6* was higher in vitellogenic than in non-vitellogenic ovaries (Levi et al., 2012), although in trout the highest *stra6* levels were recorded in ovaries of juvenile fish (Levi et al., 2008). During vitellogenesis progression, we found a significant decrease of *rbp4 and stra6* expression, coinciding with the natural increase in E_2_ plasma levels in this species (Mañanós et al., 1997; Prat et al., 1990; Rocha et al., 2009). Since vitellogenin synthesis is regulated by E_2_ levels, it seems plausible that in the European sea bass retinoid metabolism could also be mediated by the E_2_ content.

In vertebrates, RA level is set by the balance between its synthesis by Aldhs and its degradation by Cyp26 enzymes (Duester, 2008). At least three genes code for different members of the family of RA synthesizing enzymes, namely *Aldh1A1*, *Aldh1A2*, and *Aldh1A3* (Holmes, 2015). In teleosts, the general trait is the absence of *aldh1a1* (Cañestro et al., 2009; Pittlik et al., 2008) and in some species *aldh1a3* has also been lost during evolution (Feng et al., 2015; Holmes, 2015). Our results show that *aldh1a2* and *aldh1a3* are present in European sea bass, indicating that the two might be needed for the irreversible oxidation of RAL into RA in the gonads (Napoli, 2012; Rodríguez-Marí et al., 2013; Uji et al., 2011). Nevertheless, since in other teleosts *Aldh1a3* is either not expressed in the gonads (Rodríguez-Marí et al., 2013) or even absent in their genomes (Feng et al., 2015; Holmes, 2015), its role in meiosis initiation needs to be further studied.

In tetrapods, *Cyp26b1* is downregulated by the time germ cells enter meiosis, with clear sex-related differences in its timing of expression (Koubova et al., 2006; Smith et al., 2008; Wallacides et al., 2009) while in fish, *cyp26a1* seems to be a meiosis preventing factor (Feng et al., 2015; Lau et al., 2013; Li et al., 2016; Rodríguez-Marí et al., 2013). In tilapia, *cyp26a1* and *aldh1a2* are the main regulators of RA levels with high *aldh1a2* and low *cyp26a1* expression at the time of meiosis (Feng et al., 2015). Indeed, loss of gene function by CRISPR/Cas9 resulted in delayed meiosis in *aldh1a2* deficient females while meiotic initiation was advanced in *cyp26a1* deficient males, confirming the key role of both genes in the onset of meiosis in tilapia (Feng et al., 2015). Moreover, in Southern catfish (Li et al., 2016) and zebrafish (Rodríguez-Marí et al., 2013), an increase in *aldh1a2* expression was found at the time of meiosis in both sexes, further involving RA in meiosis regulation. Our results also show a clear downregulation of *cyp26a1* in early recrudescent testis, coinciding with the onset of meiosis, as previously reported by our group using a microarray expression profiling approach (Blázquez et al., 2017). This downregulation was preceded by high expression of *aldhs* in late differentiated testis, prior to the onset of meiosis. Moreover, an increase of *aldhs* concomitant with a downregulation of *cyp26a1* were found during the transition between maturation and ovulation in females, time when meiosis resumption takes place, similar to what has been reported in zebrafish (Rodríguez-Marí et al., 2013) and medaka (Adolfi et al., 2016), further implicating this enzyme in meiosis progression in the European sea bass. These studies evidence the sex-specific regulation of RA levels at the time of meiosis in different fish models. Moreover, since *cyp26a1* represents an endpoint in RA metabolism, we suggest that its downregulation in male and female gonads could be used as a suitable molecular marker for the initiation of fish meiosis.

RA synthesis and lipid metabolism are linked in a complex regulatory mechanism where RAL has a key role. Indeed, Rxr/Ppar heterodimers bind to peroxisome proliferator response elements in the promoters of different genes involved in lipid metabolism and lipogenesis, acting as transcriptional regulators (Chawla et al., 2001; Ziouzenkova and Plutzky, 2008). The present study shows that the highest expression levels of nuclear receptors (*rara*, *rxra* and *pparg*) occur right before the onset of meiosis in males, while in females, the increase during meiosis resumption is only true for *pparg*. These changes could be induced in response to the higher RA availability driven by the upregulation of *aldhs* and the decrease of *cyp26a1* during the same periods. Moreover, the increase of *pparg* expression concomitant to that of *aldh1a3* by the time of ovulation suggest a tight coordination between lipid homeostasis/energy metabolism and reproduction in European sea bass similar to what has been reported in reproductive tissues in mice (Froment et al., 2006). Indeed, a recent study showed that the lipid metabolism pathway was affected during the onset of puberty in European sea bass males, with several genes involved in the regulation of energy balance being altered, probably due to the specific energy requirements and the decrease in food intake during this reproductive stage (Blázquez et al., 2017).

Rar/Rxr heterodimers are responsible for *in vivo* RA signal transduction, using mainly atRA as ligand (Brélivet et al., 2012)*.* We also show similar expression patterns for *rara* and *rxra,* in both sexes*,* indicating their heterodimeric interdependence. The regulation of this ligand-dependent model will determine the transcription of the right RA-target gene at the precise time, suggesting a tight control of RA signaling during gonad development in European sea bass. Our data suggest that the high levels of RA synthesis (*aldh1a2*, *aldh1a3*) and of RA signaling (*rara*, *rxra, pparg*) genes found prior to the onset or by the resumption of meiosis in males and females, respectively could be triggering the transcription of other RA-induced genes involved in gonad maturation. At these stages we also found high levels of *crabp1* in both sexes consistent with its role as spatiotemporal “buffer” that protects the cell from an excess of RA, either by keeping it bound in the cytosol, facilitating its degradation by Cyp26s (Fiorella and Napoli, 1994; Won et al., 2004), or by delivering it to the nucleus where it binds to and activates RA receptors (Takase et al., 1986). Likewise, in a previous study, high *crabp1* expression was found in premeiotic European sea bass males followed by a downregulation at the time of meiosis (Blázquez et al., 2017), suggesting its role in RA availability during this process. Although Crabps are essential for hindbrain patterning in zebrafish since they compensate for changes in RA production (Cai et al., 2012), their involvement in RA signaling in mouse is not crucial (Lampron et al., 1995). In the present study, the possible increase of RA content in the gonads driven by high levels of *aldhs* in premeiotic stages might give the signal to maintain high levels of *crabp1* and *cyp26a1* in order to keep physiological intracellular RA levels and avoid possible toxic effects of an RA excess (Chung and Wolgemuth, 2004).

In females, the highest expression of RA synthesis genes, RA receptor genes, and RA binding proteins occurred in previtellogenic ovaries and decreased as vitellogenesis progressed. Expression remained low during maturation and ovulation except for *pparg* and the RA synthesis genes *aldh1a2* and *aldh1a3* that slightly increased by the time of ovulation in line with the higher RA requirements needed to complete meiosis during that developmental period. We also found high *amh* in testes prior to the onset of meiosis, in agreement with its role as a potent inhibitor of the synthesis of 11-ketotestosterone, the main androgen in fish (Borg, 1994), that characterizes the stages previous to the onset of puberty in European sea bass males (Blázquez et al., 2017; Mazón et al., 2014). Moreover, a progressive increase of estrogen levels was observed in vitellogenic ovaries, remaining high during the transition between maturation and ovulation, prior to meiosis resumption (Mañanós et al., 1997; Prat et al., 1990; Rocha et al., 2009). Altogether, our results suggest a close link between steroidogenic enzymes and RA related genes by the time of meiosis, supported by the increase of RA availability in the gonad prior to meiosis driven by the increase of *aldh1a2* and *aldh1a3* and the decrease of *cyp26a1* in both sexes. A summary figure (Supplementary Fig. 3) compiles the results on the expression of genes involved in the RA signaling pathway of European sea bass males and females.

In mice, *Stra8* is required for the onset of meiosis in embryonic ovaries (Baltus et al., 2006; Menke et al., 2003) and in juvenile testis (Anderson et al., 2008). However, this gene could have been lost in teleosts because it is absent in a number of fish genomes (Feng et al., 2015; Rodríguez-Marí et al., 2013). This was challenged by the discovery of an *stra8* ortholog in Southern catfish (Dong et al., 2013). The study stated that *stra8* could be difficult to identify in other fish species either because of a very low sequence similarity or because of incomplete genome sequencing. Our results also show an absence of *stra8* and of the conserved tetrapod genomic neighbourhood. Nevertheless, the RA signaling pathway seems to be completely functional during puberty, suggesting a role in meiosis in a *stra8* independent manner. The European sea bass genome has been sequenced to about 30x coverage using a combination of three independent sequencing technologies including whole-genome shotgun, mate pair and BAC-end sequencing (Tine et al., 2014) and recently improved with the addition of RNAseq data (Chaves-Pozo et al., 2017). It represents one of the highest-quality genomes available for an aquaculture fish species thus it seems very unlikely that the absence of *stra8* could be due to incomplete sequencing. However, it cannot be completely excluded that the low sequence similarity could be responsible for the difficulty in its identification. Recent evidence showed the presence of *stra8* homologs in several teleosts belonging to different families (Pasquier et al., 2016), but not in zebrafish or any Acanthomorpha (Rodríguez-Marí et al., 2013). The study concluded that a single *stra8* paralog was retained after the teleost-specific whole genome duplication (Hoegg et al., 2004; Taylor et al., 2003). Therefore, it was suggested that *stra8* was lost in Acanthomorpha, the largest group of teleosts where the European sea bass is included, and independently in the Cypriniform lineage due to a lineage-specific loss event (Pasquier et al., 2016).

In teleosts, RA might play a critical role in meiotic initiation through two different signaling pathways: a *stra8*-dependent and a *stra8*-independent one, although both possibilities rely on the final RA balance (Dong et al., 2013; Feng et al., 2015; Li et al., 2016). Recent evidence showed that *stra8* is not the only meiosis-inducing gene activated by RA, and experiments with *Cyp26b1* and *Stra8* mutant mice reveal *Rec8* as a new RA target gene (Koubova et al., 2014). Its regulation occurs independently but in the same temporal and spatial manner as *Stra8*. Both genes play critical roles during early meiotic processes, suggesting that RA-induced meiosis may follow two independent pathways (Koubova et al., 2014). However, further functional analyses are needed to elucidate the ultimate role of RA triggering puberty in the European sea bass.

## 5 Conclusion

To the best of our knowledge this is the first study trying to address the involvement of the RA signaling pathway in the regulation of key events during the reproductive process in male and female European sea bass. In summary, the highest levels of genes involved in the RA signaling pathway are found during the stage previous to meiosis initiation or resumption in testis and ovaries, respectively. We hypothesize that during the pre-meiotic stage, fish might be increasing the RA levels within the gonadal milieu needed to switch on meiosis by inducing an upregulation of its synthesis, transport, and signaling. *cyp26a1* levels are also kept high in order to prevent an early entry into meiosis, in agreement with the role of this enzyme as the meiosis inhibiting factor in vertebrates. The decrease of *cyp26a1* expression at the time of male meiosis and also during maturation/ovulation, when meiosis is resumed in females, will induce the increase of gonadal RA levels reaching a threshold to trigger meiosis. This decrease of *cyp26a1* could be used as a molecular marker for the onset of meiosis in European sea bass. Taken together our results suggest an increase of RA bioavailability and a subsequent signaling through nuclear receptors by the time of meiosis. The absence of *stra8* and a conserved genomic neighbourhood in the European sea bass genome suggests that RA signaling in this species does not occur through the transduction of this particular meiosis gatekeeper, as it has been demonstrated in tetrapods and other fish.

## Declarations

### Author contribution statement

Paula Medina: Performed the experiments; Analyzed and interpreted the data; Wrote the paper.

Ana Gómez, Silvia Zanuy: Conceived and designed the experiments; Analyzed and interpreted the data; Contributed reagents, materials, analysis tools or data.

Mercedes Blázquez: Conceived and designed the experiments; Performed the experiments; Analyzed and interpreted the data; Contributed reagents, materials, analysis tools or data; Wrote the paper.

### Funding statement

This work was supported by MICINN projects (AGL2011-28890) to Ana Gómez and (AGL2015-67477-C2-2-R) to Mercedes Blázquez, and from the Generalitat Valenciana (Prometeo II/2014/051) to Silvia Zanuy. Paula Medina was supported by a grant from the University of Antofagasta MECE2 (ANT0806).

### Competing interest statement

The authors declare no conflict of interest.

### Additional information

No additional information is available for this paper.

## Appendix A Supplementary data

Supplementary methodology (Medina et al) Supplementary methodology (Medina et al)

Supplemental figure S1 (Medina et al)_version1 **Supplementary Fig. S1**. Expression levels of marker genes of sex differentiation in histologically undifferentiated European sea bass gonads at 150 dph. (A) gonadal aromatase (*cyp19a1a*), (B) anti-müllerian hormone (*amh*). Samples were analysed by quantitative real-time fluorescent PCR. Expression data are shown as the normalized relative quantities of six samples run in triplicate and normalized to those of the constitutively expressed *18S* rRNA gene previously corrected with the expression of *ef1a* amplified from the same reverse transcribed template. Data are represented in box-and-whisker plots where boxes represent upper and lower quartiles and whiskers show maximum and minimum values. The horizontal line represents the median (2.56 for females and 0.33 for males for *cyp19a1a*, and 0.35 for females and 6.31 for males for *amh*) and the cross inside the box the average of the group (2.41 for females and 0.54 for males for *cyp19a1a*, and 0.4 for females and 5.95 for males for *amh*). Circles inside the plots represent expression data from each individual fish. Different letters denote statistical differences between both groups after a student t-test (*p* < 0.05). Supplemental figure S1 (Medina et al)_version1

Supplemental figure S2 (Medina et al)_version1 **Supplementary Fig. S2**. Gene expression levels of anti-müllerian hormone (*amh*) during early stages of sexual maturation in European sea bass males. Samples were analysed by quantitative real-time fluorescent PCR. Expression data are shown as the normalized relative quantities of six samples run in triplicate and normalized to those of the constitutively expressed *18S* rRNA gene previously corrected with the expression of *ef1a* amplified from the same reverse transcribed template. Data are represented in box-and-whisker plots where boxes represent upper and lower quartiles and whiskers show maximum and minimum values. The horizontal line represents the median (31.79 for late differentiated testis and 9.88 for early recrudescent testis) and the cross inside the box the average of the group (32.89 for late differentiated testis and 10.12 for early recrudescent testis). Circles inside the plots represent expression data from each individual fish. Different letters denote statistical differences between both groups after a student t-test (*p* < 0.05). Supplemental figure S2 (Medina et al)_version1

Supplemental figure S3 (Medina et al)_version1 **Supplementary Fig. S3**. Schematic diagram including a simplified summary of the results on the expression levels of key genes involved in the RA signaling pathway during the first reproductive cycle in European sea bass males and females. For each given gene, upright red arrows indicate upregulation, horizontal yellow arrows indicate no change and downright green arrows indicate downregulation. Black arrows stand for the different sampling points within each representative developmental period. The change in expression for every gene in a given developmental stage is calculated by comparison with the preceding stage. During sex differentiation, the comparisons are done between the undifferentiated and the early differentiated stages. UnT = undifferentiated testis, EdT = early differentiated testis, LdT = late differentiated testis, ErT = early recrudescent testis, UnO = undifferentiated ovary, EdO = early differentiated ovary. Supplemental figure S3 (Medina et al)_version1

Supplementary Table S1 (Medina et al) Supplementary Table S1 (Medina et al)

Supplementary table S2 (Medina et al)_version1 Supplementary table S2 (Medina et al)_version1

Supplementary table S3 (Medina et al) Supplementary table S3 (Medina et al)
