# Supplementary material for: Involvement of the retinoic acid signaling pathway in sex differentiation and pubertal development in the European sea bass Dicentrarchus labrax
Source: Heliyon. 2019 Feb 5;5(2):e01201. doi: 10.1016/j.heliyon.2019.e01201 (PMC6365411; doi:10.1016/j.heliyon.2019.e01201)
Supplement: Supplementary methodology (Medina et al) [file mmc1.docx]

**Supplementary methodology**

Data from the real-time qPCR assays were analysed using a modification of the classic ΔΔCt method that takes into account multiple reference genes and gene specific amplification efficiencies (Hellemans et al., 2007). This method has been supported by studies showing that appropriate normalization of RT-qPCR data with several stably expressed reference genes is essential to ensure accurate and reliable results (Bustin et al., 2009; Chang et al., 2010; Vandesompele et al., 2002). This model transforms quantification cycles into quantities using an exponential function based on the efficiency of the PCR reaction (for further details and formulas see Hellemans et al., 2007). The standard deviation (SD) and the coefficients of variation (CV) of the Cq values of *18s rRNA* and *ef1a*, the reference genes chosen for the study previously shown to be stable in this species (Mitter et al., 2009), were calculated for each sex and also for the total set of samples (including both sexes) to determine the most stable one (Pfaffl et al., 2004).

| Gene | Males | | Females | | Both sexes | |
| --- | --- | --- | --- | --- | --- | --- |
|  | SD | CV% | SD | CV% | SD | CV% |
| *18S rRNA* | 0.58 | 3.63 | 0.88 | 5.15 | 0.90 | 5.40 |
| *ef1a* | 1.20 | 5.76 | 1.40 | 6.87 | 1.31 | 6.34 |

Although both are suitable reference genes, *18S rRNA* was chosen as the main one since it consistently exhibited lower SD and CV values than *ef1a* in all conditions tested.

**Normalization factor using *18s rRNA* and *ef1a* as reference genes (Box1)**

A ΔCq equation was used to determine the *18s rRNA* relative quantity for each sample, using *ef1a* as reference gene. The *18S rRNA* relative quantity (RQ) was then calculated using *18S rRNA* efficiency (*E*) and the corresponding ΔCq value (Hellemans et al., 2007; Formulas 9-12). Finally, the normalization factor was calculated as the geometric mean of *18S rRNA* RQ values of every sample and subsequently used for PCR corrections (Hellemans et al., 2007; Formulas 13-14).

**Box 1**

| $\Delta Cq=Cq (18S rRNA)-Cq (ef1a)$  $18S rRNA RQ={\Delta\mathrm{Cq}}^{(E 18S rRNA)}$  $18S rRNA NF= Geometric mean 18S rRNA RQ (all samples included)$ |
| --- |

**Correction factor for each sample and corrected data (Box 2)**

Data normalization of the target gene (tg) was obtained by dividing its relative quantity (tg RQ) by the reference gene normalization factor (calculated in box 1). A target gene specific correction factor (tg CF) was also calculated by taking into account the geometric mean of the normalized relative quantities (NRQ) from every sample. Finally, the corrected data was obtained by dividing the NRQ of the tg by the correction factor for each tg (tg CRQ). Final data were expressed as normalized relative quantities calculated as the mean of all samples of the same sex at each gonad developmental stage.

**Box 2**

| $\Delta Cq=Cq (18S rRNA)-Cq (tg)$  $tg RQ={\Delta\mathrm{Cq}}^{(E tg)}$  $tg NRQ=\frac{(tg RQ)}{(18S rRNA NF)}$  $tg CF= Geometric mean tg NRQ (all samples included)$  $tg CRQ=\frac{(tg NRQ)}{(tg CF)}$ |
| --- |

**References**

Bustin, S.A., Benes, V., Garson, J.A., Hellemans, J., Huggett, J., Kubista, M., Mueller, R., Nolan, T., Pfaffl, M.W., Shipley, G.L., 2009. The MIQE guidelines: minimum information for publication of quantitative real-time PCR experiments. Clin. Chem. 55, 611-622.

Chang, K.H., Mestdagh, P., Vandesompele, J., Kerin, M.J., Miller, N., 2010. MicroRNA expression profiling to identify and validate reference genes for relative quantification in colorectal cancer. BMC cancer 10, 173.

Hellemans, J., Mortier, G., De Paepe, A., Speleman, F., Vandesompele, J., 2007. qBase relative quantification framework and software for management and automated analysis of real-time quantitative PCR data. Genome Biol. 8, R19.

Mitter, K., Kotoulas, G., Magoulas, A., Mulero, V., Sepulcre, P., Figueras, A., Novoa, B., Sarropoulou, E., 2009. Evaluation of candidate reference genes for QPCR during ontogenesis and of immune-relevant tissues of European seabass (*Dicentrarchus labrax*). Comp. Biochem. Physiol. B: Biochem. Mol. Biol. 153, 340-347.

Pfaffl, M.W., Tichopad, A., Prgomet, C., Neuvians, T.P., 2004. Determination of stable housekeeping genes, differentially regulated target genes and sample integrity: BestKeeper–Excel-based tool using pair-wise correlations. Biotech. Lett. 26, 509-515.

Vandesompele, J., De Preter, K., Pattyn, F., Poppe, B., Van Roy, N., De Paepe, A., Speleman, F., 2002. Accurate normalization of real-time quantitative RT-PCR data by geometric averaging of multiple internal control genes. Genome Biol. 3, research0034. 0031.
