# Supplementary figures and images for: Involvement of the retinoic acid signaling pathway in sex differentiation and pubertal development in the European sea bass Dicentrarchus labrax
Source: Heliyon. 2019 Feb 5;5(2):e01201. doi: 10.1016/j.heliyon.2019.e01201 (PMC6365411; doi:10.1016/j.heliyon.2019.e01201)

## Slide 1
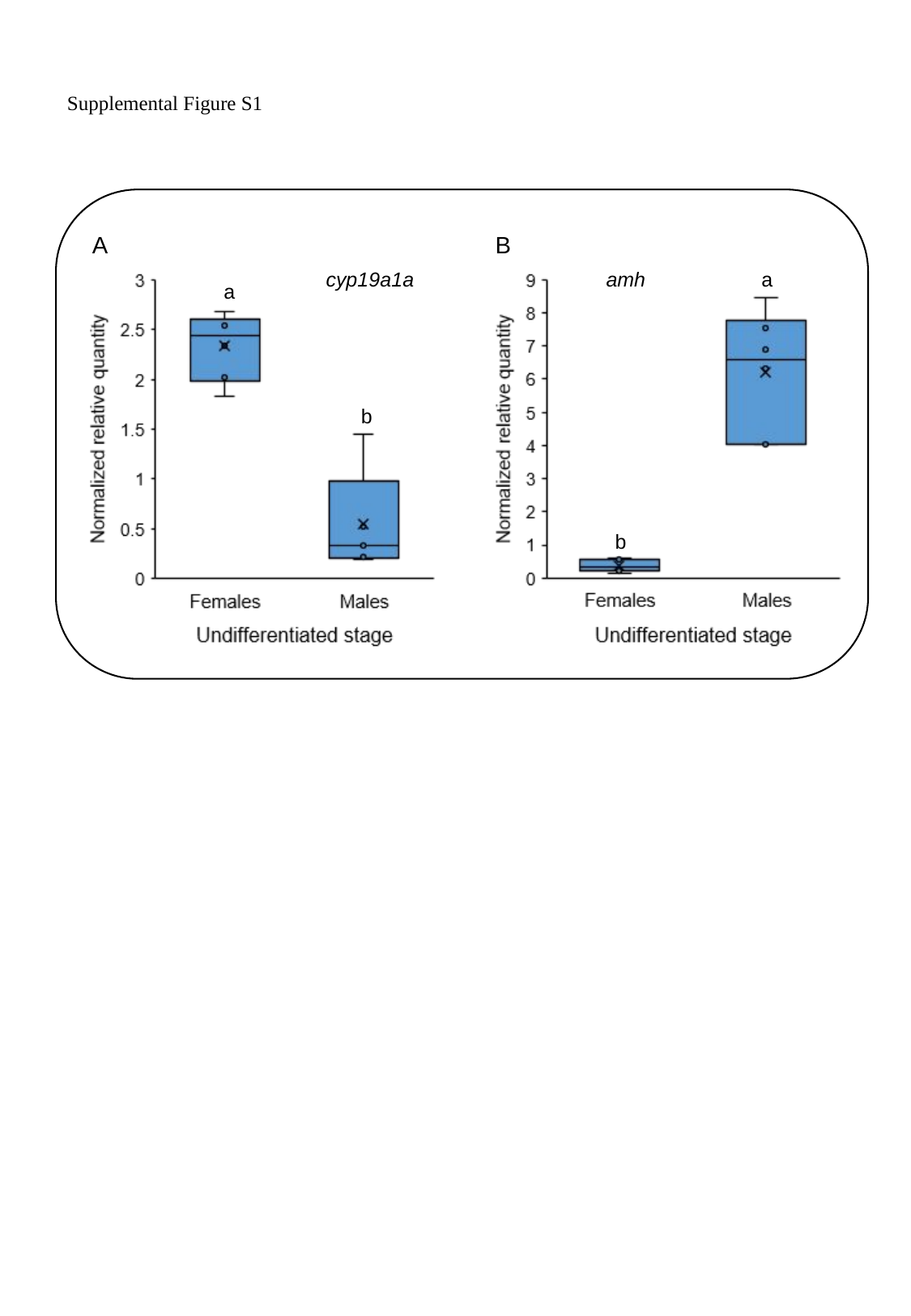

Supplemental Figure S1
A
B
cyp19a1a
a
amh
a
b
b

Supplement: Supplemental figure S1 (Medina et al)_version1 — Supplementary Fig. S1. Expression levels of marker genes of sex differentiation in histologically undifferentiated European sea bass gonads at 150 dph. (A) gonadal aromatase (cyp19a1a), (B) anti-müllerian hormone (amh). Samples were analysed by quantitative real-time fluorescent PCR. Expression data are shown as the normalized relative quantities of six samples run in triplicate and normalized to those of the constitutively expressed 18S rRNA gene previously corrected with the expression of ef1a amplified from the same reverse transcribed template. Data are represented in box-and-whisker plots where boxes represent upper and lower quartiles and whiskers show maximum and minimum values. The horizontal line represents the median (2.56 for females and 0.33 for males for cyp19a1a, and 0.35 for females and 6.31 for males for amh) and the cross inside the box the average of the group (2.41 for females and 0.54 for males for cyp19a1a, and 0.4 for females and 5.95 for males for amh). Circles inside the plots represent expression data from each individual fish. Different letters denote statistical differences between both groups after a student t-test (p < 0.05). [file mmc2.pptx]

## Slide 1
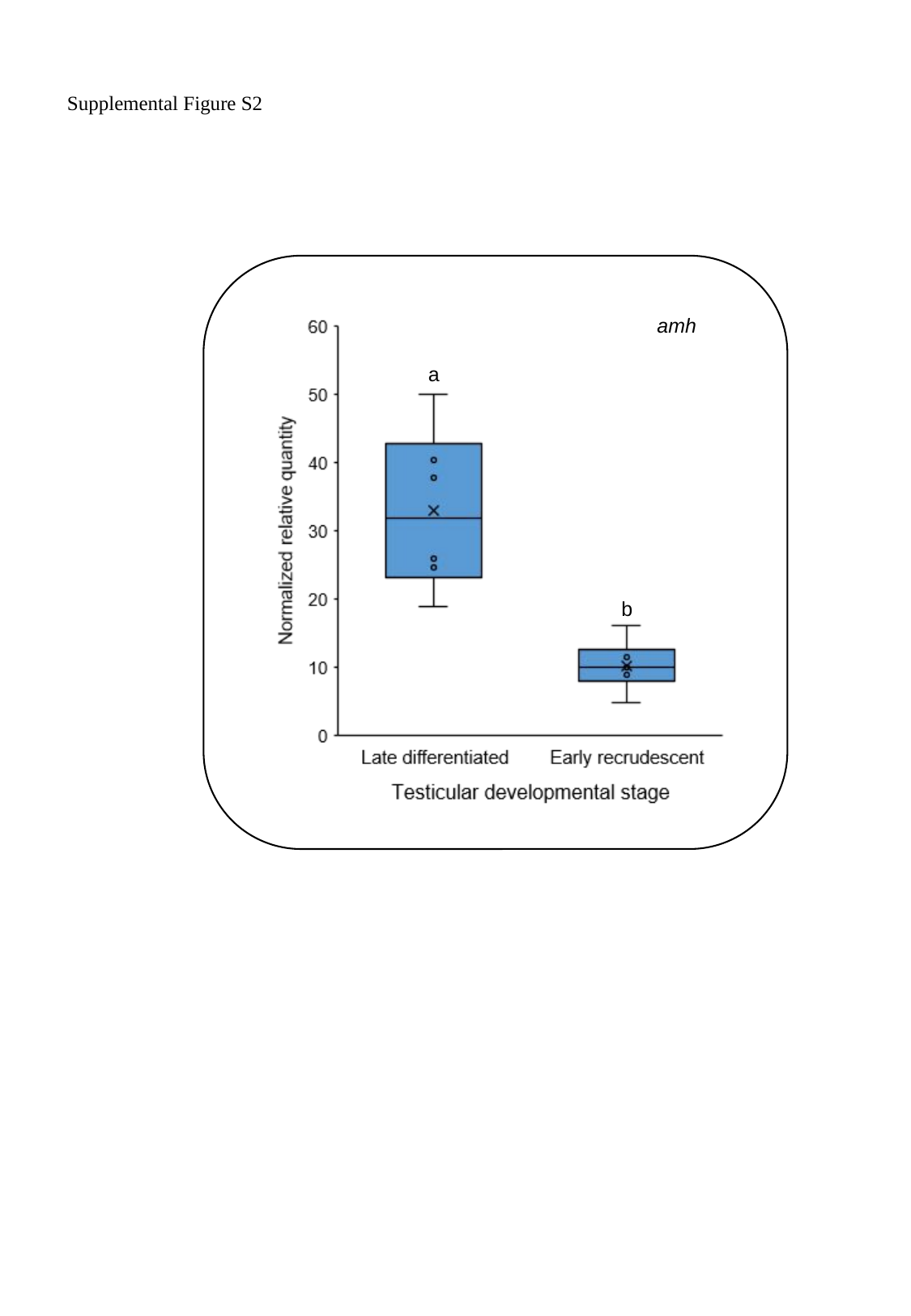

Supplemental Figure S2
amh
a
b

Supplement: Supplemental figure S2 (Medina et al)_version1 — Supplementary Fig. S2. Gene expression levels of anti-müllerian hormone (amh) during early stages of sexual maturation in European sea bass males. Samples were analysed by quantitative real-time fluorescent PCR. Expression data are shown as the normalized relative quantities of six samples run in triplicate and normalized to those of the constitutively expressed 18S rRNA gene previously corrected with the expression of ef1a amplified from the same reverse transcribed template. Data are represented in box-and-whisker plots where boxes represent upper and lower quartiles and whiskers show maximum and minimum values. The horizontal line represents the median (31.79 for late differentiated testis and 9.88 for early recrudescent testis) and the cross inside the box the average of the group (32.89 for late differentiated testis and 10.12 for early recrudescent testis). Circles inside the plots represent expression data from each individual fish. Different letters denote statistical differences between both groups after a student t-test (p < 0.05). [file mmc3.pptx]
