# Supplementary material for: Involvement of the retinoic acid signaling pathway in sex differentiation and pubertal development in the European sea bass Dicentrarchus labrax
Source: Heliyon. 2019 Feb 5;5(2):e01201. doi: 10.1016/j.heliyon.2019.e01201 (PMC6365411; doi:10.1016/j.heliyon.2019.e01201)
Supplement: Supplemental figure S3 (Medina et al)_version1 — Supplementary Fig. S3. Schematic diagram including a simplified summary of the results on the expression levels of key genes involved in the RA signaling pathway during the first reproductive cycle in European sea bass males and females. For each given gene, upright red arrows indicate upregulation, horizontal yellow arrows indicate no change and downright green arrows indicate downregulation. Black arrows stand for the different sampling points within each representative developmental period. The change in expression for every gene in a given developmental stage is calculated by comparison with the preceding stage. During sex differentiation, the comparisons are done between the undifferentiated and the early differentiated stages. UnT = undifferentiated testis, EdT = early differentiated testis, LdT = late differentiated testis, ErT = early recrudescent testis, UnO = undifferentiated ovary, EdO = early differentiated ovary. [file mmc4.pptx]

## Slide 1
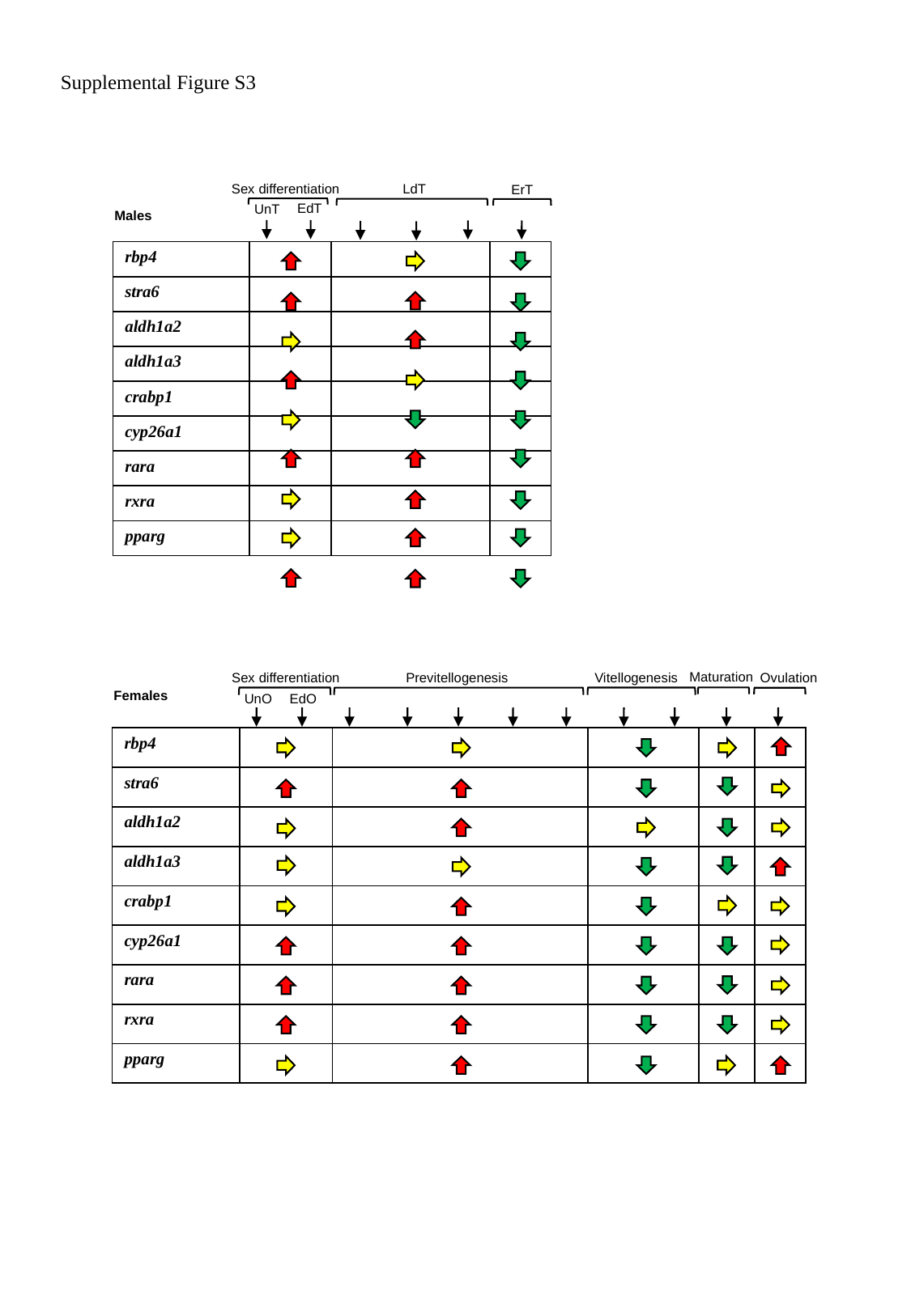

Supplemental Figure S3
Sex differentiation
LdT
ErT
EdT
UnT
Males
| rbp4 | | | |
| --- | --- | --- | --- |
| stra6 | | | |
| aldh1a2 | | | |
| aldh1a3 | | | |
| crabp1 | | | |
| cyp26a1 | | | |
| rara | | | |
| rxra | | | |
| pparg | | | |
Maturation
Sex differentiation
Ovulation
Previtellogenesis
Vitellogenesis
Females
UnO
EdO
| rbp4 | | | | | |
| --- | --- | --- | --- | --- | --- |
| stra6 | | | | | |
| aldh1a2 | | | | | |
| aldh1a3 | | | | | |
| crabp1 | | | | | |
| cyp26a1 | | | | | |
| rara | | | | | |
| rxra | | | | | |
| pparg | | | | | |
