# Supplementary material for: Involvement of the retinoic acid signaling pathway in sex differentiation and pubertal development in the European sea bass Dicentrarchus labrax
Source: Heliyon. 2019 Feb 5;5(2):e01201. doi: 10.1016/j.heliyon.2019.e01201 (PMC6365411; doi:10.1016/j.heliyon.2019.e01201)
Supplement: Supplementary Table S1 (Medina et al) [file mmc5.doc]

Supplemental table S1. Quantitative real time (qPCR) primers and features to calculate the efficiency (*E*)

| Gene | Accession no. | Primer sequence (5’→3’)^a^ | Size (bp) | Slope | *E* | R^2^ |
| --- | --- | --- | --- | --- | --- | --- |
| *rbp4* | KP739863 | F: ACCCTGCCAAGTTCAGAATG  R: GTGGACGGCGTAGTTATCGT | 107 | -3.44 | 1.95 | 1.03 |
| *crabp1* | KP723829 | F: GCCACTTGGGAAACAGAAAA  R: CATCGGCTCCAAAGATCAGT | 124 | -3.36 | 1.98 | 1.01 |
| *aldh1a2* | *D.labrax* genome | F: GCAGACAAGGCTGATGTTGA  R: TTCCTTGGAGGTCCACAAAC | 214 | -3.31 | 2.01 | 0.99 |
| *aldh1a3* | *D.labrax* genome | F: CCTGGTGTCAGCAGTCTT  R: ACCCTCCAAAGGGAGTCTGT | 125 | -3.19 | 2.06 | 0.96 |
| *stra6* | *D.labrax* genome | F: CTTGTCATGGACGCACTTTG  R: AAGGAATGCCAAGCAAGCTA | 124 | -3.92 | 1.80 | 1.18 |
| *rara* | KP749835 | F: CGCTAAACCGAACCCAGA  R: CTTCTCGGCCTGTTCCAA | 170 | -3.28 | 2.02 | 0.99 |
| *rxra* | KP749834 | F: CTGGTAGAGTGGGCCAAGAG  R: GTTCTGTGAGCACCCTGTCA | 226 | -3.43 | 1.96 | 1.03 |
| *pparg* | AY590303 | F: CAGATCTGAGGGCTCTGTCC  R: CCTGGGTGGGTATCTGCTTA | 186 | -3.46 | 1.94 | 1.04 |
| *cyp26a1* | KJ1876578 | F: GCAGGAGCTGGTGGAAGCTT  R: CCTTGCCTTCAGACCCCTGTA | 120 | -3.29 | 2.01 | 0.99 |
| *pcna* | JQ755266 | F: CCAAGGACGGAGTCAAGTTC  R: CTGGACGGGTTCATTCATCT | 125 | -3.37 | 1.98 | 1.01 |
| *cyp19a1* | AJ311177 | F: AGACAGCAGCCCAGGAGTTG  R: TGCAGTGAAGTTGATGTCCAGTT | 106 | -3.30 | 2.01 | 0.99 |
| *amh* | AM232701 | F: TGACTCCACTTCTGCTTTTCTCAT  R: AGAAAGGAGGAGGTCTGTGAAGAG | 100 | -3.68 | 1.87 | 1.11 |
| *r18s* | AY831388 | F: CCGCTTTGGTGACTCTAGATAACC  R: CAGAAAGTACCATCGAAAGTTGATAGG | 110 | -3.30 | 2.00 | 0.99 |
| *ef1a* | AJ866727 | F: AGATGCACCACGAGTCTCTGC  R: CTTGGGTGGGTCGTTCTTG | 128 | -3.27 | 2.02 | 0.98 |

^a^ Forward (F) and reverse (R) primers were obtained from Invitrogen
