# Supplementary material for: Involvement of the retinoic acid signaling pathway in sex differentiation and pubertal development in the European sea bass Dicentrarchus labrax
Source: Heliyon. 2019 Feb 5;5(2):e01201. doi: 10.1016/j.heliyon.2019.e01201 (PMC6365411; doi:10.1016/j.heliyon.2019.e01201)
Supplement: Supplementary table S2 (Medina et al)_version1 [file mmc6.docx]

Supplemental Table S2. Ensembl accession numbers used for *stra8* synteny analysis.

| **Species (Abreviation)** | **Ensembl database** | **STRA8 ID number** |
| --- | --- | --- |
| Homo sapiens (Hsa) | GRCh38 | ENSG00000146857 |
| Mus musculus (Mmu) | GRCm38.p3 | ENSMUSG00000029848 |
| Taenopygia guttata (Tgut) | taeGut3.2.4 | ENSTGUG00000009183 |
| Pelodiscus sinensis (Psi) | PelSin_1.0 | ENSPSIG00000013824 |
| Gallus gallus (Gga) | Galgal4 | ENSGALG00000011722 |
| Xenopus tropicalis (Xtr) | JGI4.2 | ENSXETG00000020235 |
| Lepisosteus oculatus (Loc) | LepOcu1 | ENSLOCG00000016111 |
| Tetraodon nigroviridis (Tni) | TETRAODON8.0 | Gene not found |
| Gasterosteus aculeatus (Gac) | BROADS1 | Gene not found |
| Danio rerio (Dre) | Zv9 | Gene not found |
| Dicentrarchus labrax (Dla) | dicLab v1.0c | Gene not found |
