# Supplementary material for: Involvement of the retinoic acid signaling pathway in sex differentiation and pubertal development in the European sea bass Dicentrarchus labrax
Source: Heliyon. 2019 Feb 5;5(2):e01201. doi: 10.1016/j.heliyon.2019.e01201 (PMC6365411; doi:10.1016/j.heliyon.2019.e01201)
Supplement: Supplementary table S3 (Medina et al) [file mmc7.docx]

Supplemental Table S3. Genes located in *Stra8* genomic neighbourhood

| Gene name | Gene symbol | Function |
| --- | --- | --- |
| phosphodiesterase 6H | *pde6h* | Involved in the transmission and amplification of the visual signal |
| ATP/GTP binding protein like 3 | *agbl3* | Codes for a metallocarboxypeptidase that mediates deglutamylation and deaspartylation of target proteins such as tubulins. |
| transmembrane protein 140 | *tmem140* | Codes for a transmembrane protein and contains transcription factor binding sites for pparg in its promoter |
| WD Repeat Domain 91 | *wdr91* | Mutations in this gene are associated with retinitis pigmentosa, a degenerative disease that causes vision impairment |
| CCR4-NOT transcription complex subunit 4 | *cnot4* | Involved in protein ubiquitination |
| Nucleoporin 205 | *nup205* | Encodes a subunit of the nuclear pore complex involved in the active transport of proteins and RNAs between the nucleus and cytoplasm |
| ADP ribosilation factor like GTPase 1 | *arl1* | Encodes a protein involved in the regulation of intracellular vesicular membrane trafficking. Important for the function of the Golgi apparatus |
| solute carrier family 13 member 4 | *slc13a4* | Involved in the transport of glucose and other sugars, bile salts and organic acids, metal ions and amine compounds |
| family with sequence similarity 180 member A | *fam180A* | Osteoblastic specific gene |
| UTP20, small subunit processome component | *utp20* | Involved in 18S pre-rRNA processing |
